# Selective enhancement of fear extinction by inhibiting neuronal adenylyl cyclase 1 (AC1) in aged mice

**DOI:** 10.1186/s13041-024-01083-9

**Published:** 2024-02-22

**Authors:** Wantong Shi, Qi-Yu Chen, Yujie Ma, Jinjin Wan, Xu-Hui Li, Min Zhuo

**Affiliations:** 1 Center for Neuron and Disease, Frontier Institutes of Science and Technology, Xi’an Jiaotong University, Xi’an, Shaanxi China; 2 Zhuomin Institute of Brain Research, Qingdao, Shandong China; 3 CAS Key Laboratory of Brain Connectome and Manipulation, Interdisciplinary Center for Brain Information, Chinese Academy of Sciences Shenzhen Institute of Advanced Technology, Shenzhen, Guangdong China; 4 Oujiang Laboratory, Wenzhou Medical University, Wenzhou, Zhejiang China; 5 Department of Physiology, Faculty of Medicine, University of Toronto, Medical Science Building, 1 King’s College Circle, Toronto, ON M5S 1A8 Canada

**Keywords:** hNB001, AC1, Trace fear memory, Remote fear memory, Extinction

## Abstract

Adenylyl cyclase 1 (AC1) is a selective subtype of ACs, which is selectively expressed in neurons. The activation of AC1 is activity-dependent, and AC1 plays an important role in cortical excitation that contributes to chronic pain and related emotional disorders. Previous studies have reported that human-used NB001 (hNB001, a selective AC1 inhibitor) produced analgesic effects in different animal models of chronic pain. However, the potential effects of hNB001 on learning and memory have been less investigated. In the present study, we found that hNB001 affected neither the induction nor the expression of trace fear, but selectively enhanced the relearning ability during the extinction in aged mice. By contrast, the same application of hNB001 did not affect recent, remote auditory fear memory, or remote fear extinction in either adult or aged mice. Furthermore, a single or consecutive 30-day oral administration of hNB001 did not affect acute nociceptive response, motor function, or anxiety-like behavior in either adult or aged mice. Our results are consistent with previous findings that inhibition of AC1 did not affect general sensory, emotional, and motor functions in adult mice, and provide strong evidence that inhibiting the activity of AC1 may be beneficial for certain forms of learning and memory in aged mice.

## Introduction

The adenylyl cyclase 1 (AC1) plays a critical role in pain-related plasticity in the anterior cingulate cortex (ACC), a key cortical area that contributes to pain perception and emotional responses [1–3]. Long-term potentiation (LTP) is a major cellular model for understanding chronic pain and fear memory neural mechanisms [1]. In the ACC of AC1 knock-out (KO) mice, the induction of both presynaptic and postsynaptic LTP was blocked [3–5]. Behavioral sensitization in animal models of inflammatory pain was blocked, while acute pain is normal in AC1 KO mice [6]. In addition, pharmacological inhibition of the AC1 by a selective AC1 inhibitor, NB001, has been reported to produce powerful analgesic effects on different animal models of chronic pain including neuropathic pain, inflammatory pain, cancer pain, arthralgia, gout-related pain, visceral pain, and headache [7–12]. Consistent with those found in AC1 KO mice, NB001 did not significantly affect sensory, motor, or emotional responses in adult animals [7–12].

Hippocampal LTP is important for certain forms of learning and memory [13]. Many protein kinases have been reported to contribute to learning-related LTP, including calcium/calmodulin-dependent protein kinase type II (CaMKII), protein kinase C (PKC), protein kinase A (PKA), the tyrosine kinase Src, and mitogen-activated protein kinase (MAPK) [13, 14]. The Ca^2+^-stimulated ACs, AC1 and AC8, also play critical roles in late-phase LTP (L-LTP) and behavioral memory. While single genetic deletion of AC1 or AC8 did not cause any significant changes in synaptic LTP in the hippocampus and behavioral memory, double knockout of both AC1 and AC8 produced the reduction of hippocampal L-LTP and spatial memory in adult mice [15]. Therefore, the AC1 activity may be compensated by AC8, or other signaling molecules (such as various protein kinases) that are crucial for hippocampal LTP and memory [16–18]. Interestingly, genetic overexpression of AC1 in the forebrain of adult mice enhanced recognition memory and hippocampal LTP [19]. This is in good accordance with a previous work on NMDA receptor GluN2B overexpression in the forebrain, since the AC1 signaling pathway acts as the downstream of GluN2B containing NMDA receptors [20, 21]. However, in aged animals, it has been reported that the expression of AC1 mRNA was downregulated in the hippocampus [22]. One direct way to rescue memory impairment in aged animals is genetic overexpression of AC1 in the forebrain including the hippocampus. However, opposite results have been found that overexpression of AC1 in the forebrain in aged mice impaired spatial memory [23], suggesting that AC1 activity may not be beneficial for learning and memory in aged animals.

Previous studies used genetic manipulation to enhance the AC1 activity [23]. It is difficult to rule out other developmental-related changes and compensation (such as AC8) after AC1 overexpression. In the present study, we used a selective AC1 inhibitor to examine if the AC1 activity is critical for memory in aged mice. Human-used NB001 (hNB001), which is found to be safe in healthy human subjects [24], was used in both adult and aged mice. We investigated the potential behavioral effects of a single or long-term oral administration of hNB001 in adult and aged mice.

## Results

### hNB001 enhanced relearning of trace fear in aged mice

**Fig. 1:**
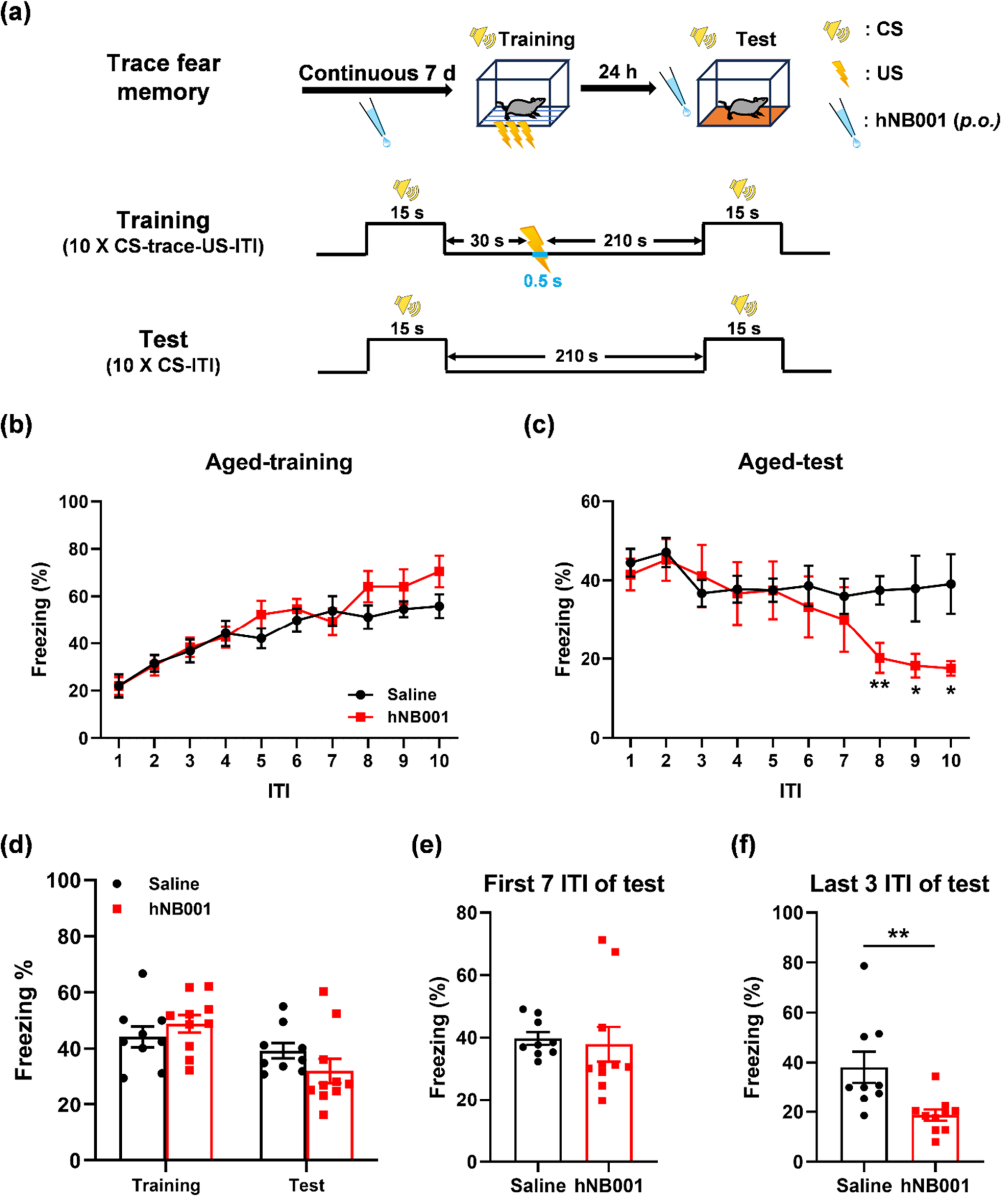
Effects of hNB001 on trace fear memory in aged mice. **a** Schematic diagram showing trace fear memory performed on aged mice. The CS of a white noise (80 dB, 15 s) was delivered 30 s (trace) before the US of a foot shock (0.75 mA, 0.5 s). Mice were conditioned by 10 CS-trace-US-ITI (210 s) trials for training, and received 10 CS–ITI trials in a novel chamber for test after 24 h of training. The mice were administered hNB001 (30 mg/kg) or saline orally for 7 days before training. hNB001 or saline was taken orally 45 min before test. **b** No effects of hNB001 on trace fear conditioning in aged mice (Saline, n = 9 mice; hNB001, n = 10 mice, Two-way ANOVA, F _(1,17)_ = 0.9233, p = 0.3501). **c** hNB001-treated mice showed no significant difference in freezing during ITI-1 to ITI-7, but significantly reduced freezing during ITI-8 to ITI-10, compared with saline-treated mice during trace fear test (Two-way ANOVA, F _(1,17)_ = 1.817, p = 0.1953; ITI-8, Student’s t-test, t _(17)_ = 3.271, p = 0.0045; ITI-9, Student’s t-test, t _(17)_ = 2.316, p = 0.0333; ITI-10, Student’s t-test, t _(17)_ = 2.896, p = 0.0100). **d** Statistical results of trace fear conditioning and test in aged mice with oral hNB001 or saline (Student’s t-test, Training, t _(17)_ = 0.9609, p = 0.3501; Test, t _(17)_ = 1.348, p = 0.1953). **e** hNB001-treated mice showed no significant difference in freezing during the first 7 ITI of trace fear test, compared with saline-treated mice (Student’s t-test, t _(17)_ = 0.2986, p = 0.7689). **f** hNB001-treated mice showed significantly reduced freezing during the last 3 ITI of trace fear test, compared with saline-treated mice (Student’s t-test, t _(17)_ = 3.055, p = 0.0072). *p < 0.05, **p < 0.01

To examine the effects of AC1 inhibitor hNB001 on learning and memory, we performed the trace fear conditioning paradigm in aged mice after oral administration of hNB001 or saline. This paradigm differs from the classic delay fear conditioning paradigm. Trace fear conditioning has a trace interval between the conditioned stimulus (CS) and the unconditioned stimulus (US). The animal must maintain attention during the trace interval to learn the CS-US association [25]. The CS was an 80 dB white noise for 15 s. The US was a 0.75-mA electric footshock for 0.5 s. Trace fear training introduced a 30 s time interval (trace) between the CS and the US. Mice were conditioned by 10 CS-trace-US-intertrial interval (ITI, 210 s) trials for training after oral administration of hNB001 (30 mg/kg) or saline for 7 days. Mice received 10 CS-ITI trials in a novel chamber for testing after 24 h of training [26] (Fig. 1a).

Saline-treated mice displayed increased freezing throughout the training session (ITI-1 vs ITI-10), suggesting successfully learned after trace fear conditioning. hNB001-treated aged mice also showed increased freezing after trace fear conditioning (Fig. 1b). There was no significant difference between the two groups in freezing of per ITI or average freezing of all ITIs (Fig. 1b, d). During the test of trace fear, there was no significant difference in freezing from ITI-1 to ITI-7 between the two groups (Fig. 1c, e). However, hNB001-treated mice displayed significantly reduced freezing from ITI-8 to ITI-10 compared with saline-treated mice (Fig. 1c, f). During the test phase, hNB001-treated mice relearned that the CS was no longer associated with the footshock, experienced fear extinction, and this new inhibitory learning could suppress previously established memory. Taken together, these results suggest that oral administration of hNB001 did not affect the acquisition of trace fear memory during training, or the expression of trace fear memory during testing in aged mice. However, oral administration of hNB001 enhanced the relearning of trace fear in aged mice.

### Effects of hNB001 on recent and remote auditory fear memory in mice

**Fig. 2:**
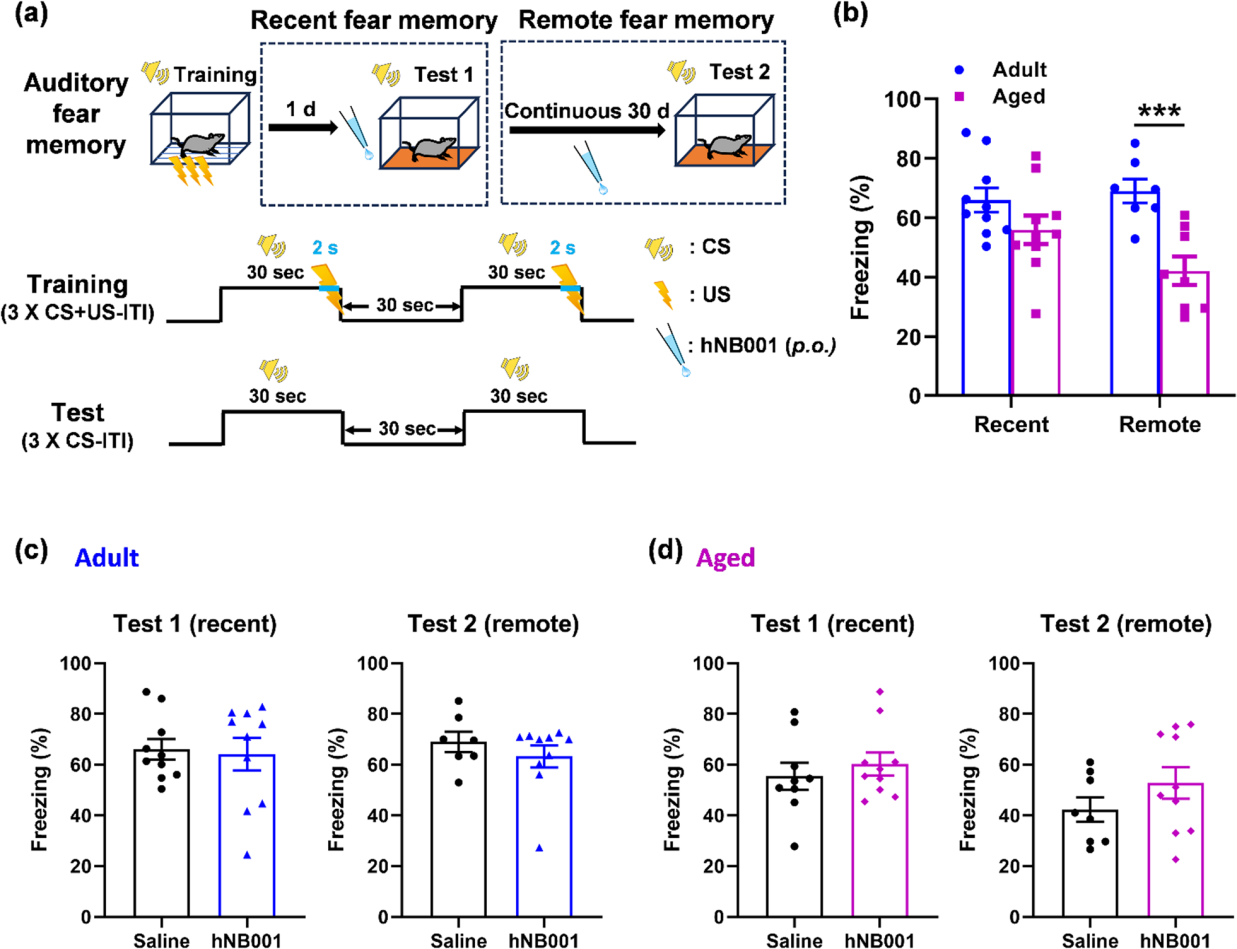
Effects of hNB001 on recent or remote auditory fear memory in adult or aged mice. **a** Schematic diagram showing auditory fear memory performed on mice. The CS is a tone (2800 Hz, 85 dB, 30 s). The US is a foot shock (0.75 mA, 2 s) that co-terminated with the tone. Mice were conditioned by three CS/US pairings at 30 s intervals for training. 45 min after oral administration of hNB001 (10 mg/kg) or saline, mice received 3 CS–ITI trials in a novel chamber for recent fear memory test after one day of training (Test 1). The mice were given hNB001 orally twice a day for 30 days. Remote fear memory test was performed again 30 days later (Test 2). **b** There was no significant difference in the recent fear memory between adult and aged mice, but remote fear memory of aged mice was significantly impaired compared with adult mice (Recent, saline, n = 10 mice, hNB001, n = 10 mice, Student’s t-test, t _(18)_ = 1.594, p = 0.1283; Remote, saline, n = 7 mice, hNB001, n = 8 mice, Student’s t-test, t _(13)_ = 4.221, p = 0.0010). **c** Oral administration of hNB001 for a single or 30 days did not affect recent (left) or remote (right) auditory fear memory in adult mice (Recent, saline, n = 10 mice, hNB001, n = 10 mice, Student’s t-test, t _(18)_ = 0.2439, p = 0.8101; Remote, saline, n = 7 mice, hNB001, n = 10 mice, Student’s t-test, t _(15)_ = 0.9273, p = 0.3685). **d** Oral administration of hNB001 for a single or 30 days did not affect recent (left) or remote (right) auditory fear memory in aged mice (Recent, saline, n = 9 mice, hNB001, n = 10 mice, Student’s t-test, t _(17)_ = 0.6973, p = 0.4950; Remote, saline, n = 8 mice, hNB001, n = 10 mice, Student’s t-test, t _(16)_ = 1.296, p = 0.2133). ***p < 0.001

Previous research found that NB001 did not significantly affect recent contextual fear memory in adult mice [7, 27]. To further verify the effects of hNB001 on recent and remote fear memory in mice, we used the auditory fear memory paradigm in mice after oral administration of hNB001 or saline. The CS was an 85-db tone at 2800 Hz for 30 s. The US was a 0.75-mA electric footshock for 2 s. The CS and the US end at the same time. Mice were conditioned by three CS-US pairing and ITI (30 s) trials for training. One day after training, mice received 3 CS-ITI trials in a novel chamber for Test 1 of recent fear memory 45 min after oral administration of hNB001 (10 mg/kg) or saline. Mice were then treated with hNB001 or saline orally twice a day for 30 days. Thirty days after training, mice received 3 CS-ITI trials in a novel chamber for Test 2 of remote fear memory [6, 28] (Fig. 2a).

First, we compared recent or remote auditory fear memory between adult and aged mice. There was no significant difference in the recent auditory fear memory between adult and aged mice, but remote auditory fear memory of aged mice was significantly impaired compared to adult mice (Fig. 2b). Next, we examined whether hNB001 affects recent and remote fear memory in adult mice. After one day of training, there was no significant difference in freezing of the recent fear memory test between hNB001- and saline-treated adult mice. Adult mice were taken orally with either hNB001 or saline twice daily for 30 days after training, there was also no significant difference in freezing of remote fear memory test between the two groups. (Fig. 2c). Furthermore, we also examined the effects of hNB001 on recent and remote fear memory in aged mice. Similarly, we found that oral injection of hNB001 did not produce a significant difference in freezing of recent fear memory test in aged mice. In the remote fear memory test, hNB001 did not cause any significant changes in the freezing of aged mice (Fig. 2d). These results illustrate that hNB001 does not affect recent or remote auditory fear memory in adult or aged mice.

### Effects of hNB001 on remote auditory fear extinction in mice

**Fig. 3:**
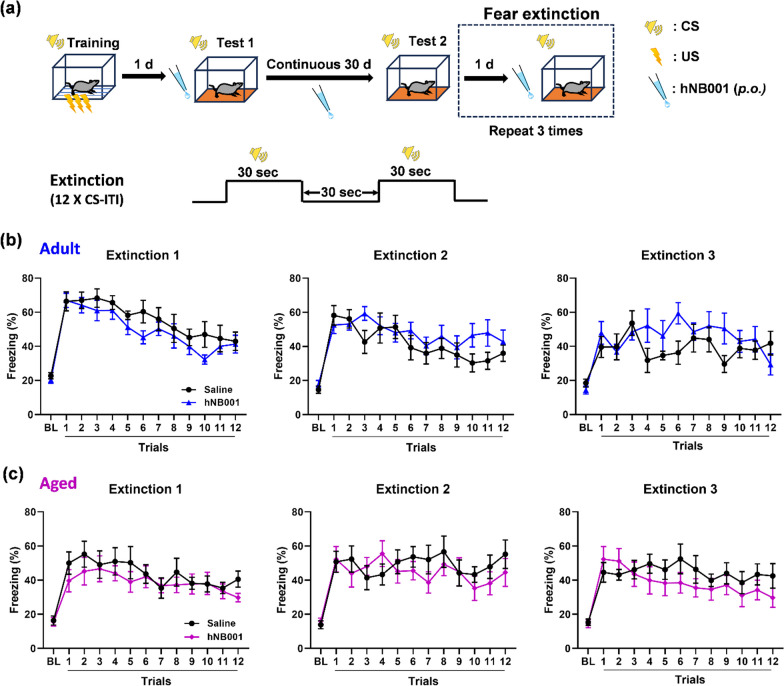
Effects of continuous administration of hNB001 on remote fear extinction in mice. **a** Schematic diagram showing fear extinction performed on mice. After auditory fear memory tests, the mice performed fear extinction (12 CS-ITI) once a day for three consecutive days. **b** hNB001 does not affect remote extinction 1/2/3 in adult mice (Saline, n = 9 mice, hNB001, n = 9 mice, Two-way ANOVA, Extinction 1, F _(1,16)_ = 1.365, p = 0.2597; Extinction 2, F _(1,16)_ = 1.792, p = 0.1993; Extinction 3, F _(1,16)_ = 1.807, p = 0.1976). **c** hNB001 does not affect remote extinction 1/2/3 in aged mice (Saline, n = 8 mice, hNB001, n = 10 mice, Two-way ANOVA, Extinction 1, F _(1,16)_ = 0.6108, p = 0.4459; Extinction 2, F _(1,16)_ = 0.2314, p = 0.6370; Extinction 3, F _(1,16)_ = 0.6563, p = 0.4298)

In the absence of the US, continuous re-exposure to the CS induced extinction. Fear memory extinction was not simply memory forgetting or disruption, but rather a form of inhibitory learning [29]. To further demonstrate the effects of hNB001 on relearning behavior, we tested remote auditory fear extinction of hNB001- or saline-treated mice. The next day after remote fear memory test, fear extinction was performed once a day for consecutive three days (Extinction 1–3). After 45 min of treatment with hNB001 (10 mg/kg) or saline, mice received 12 CS-ITI trials (CS, 85-db, 2800 Hz, 30 s; ITI, 30 s) in a novel chamber for extinction [30] (Fig. 3a). During remote fear extinction (Extinction 1/2/3), there was no significant difference in freezing between hNB001- and saline-treated adult mice (Fig. 3b). Similar with the results of adult mice, hNB001 did not affect freezing of remote fear extinction in aged mice (Fig. 3c). In summary, hNB001 does not affect remote auditory fear extinction in adult or aged mice.

### Effects of a single administration of hNB001 on nociception, motor function, and anxiety-like behavior

**Fig. 4:**
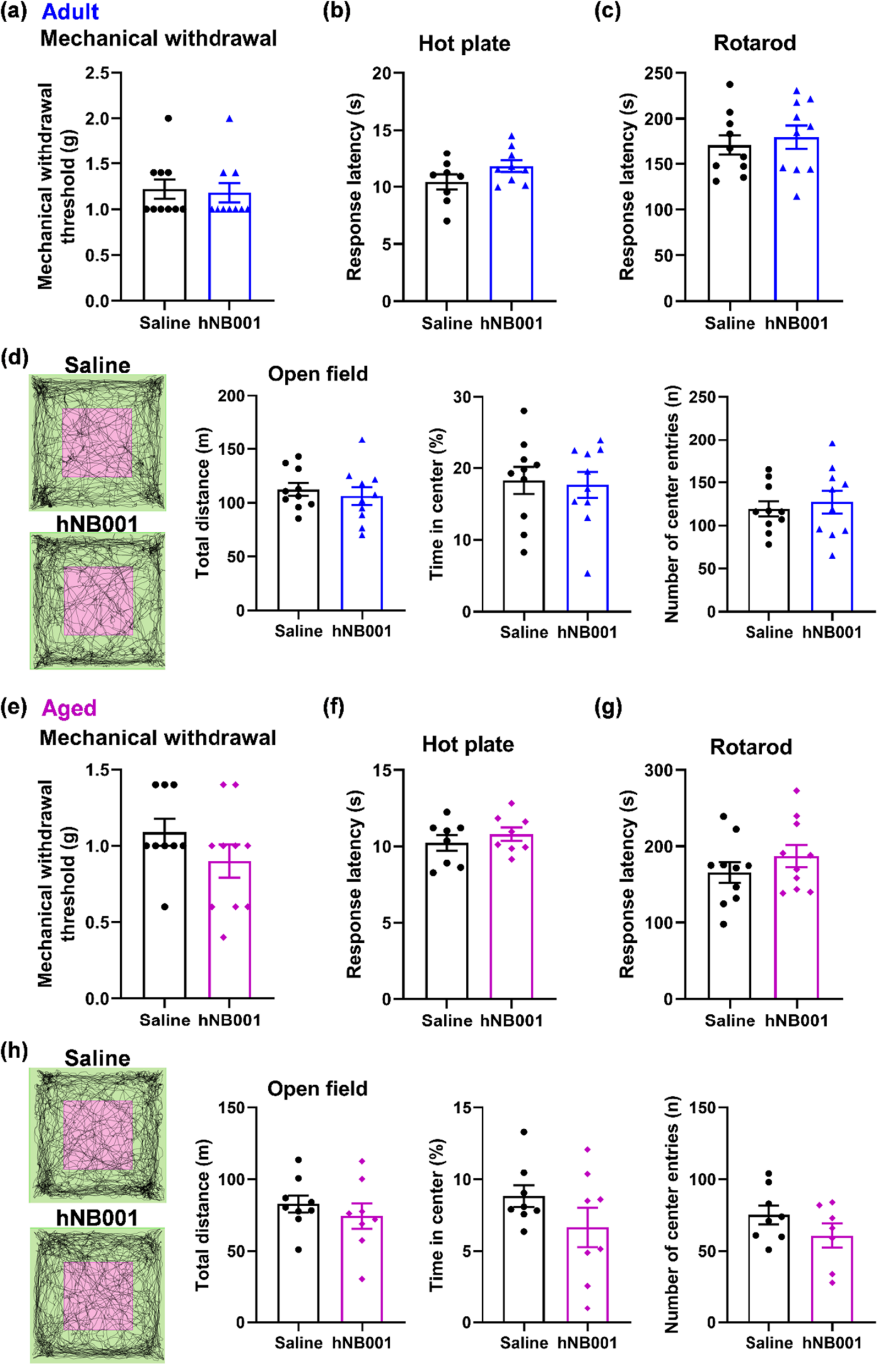
Effects of a single administration of hNB001 on nociception, motor, and anxiety-like behavior in mice. **a, b** There was no significant difference in hind paw withdrawal to von Frey filaments (**a**) and response latency of the hot plate test (**b**) after a single oral administration of 10 mg/kg hNB001 in adult mice, compared with the saline group (Mechanical withdrawal, saline, n = 10 mice, hNB001, n = 10 mice, Student’s t-test, t _(18)_ = 0.2689, p = 0.7911; Hot plate, saline, n = 8 mice, hNB001, n = 9 mice, Student’s t-test, t _(15)_ = 1.651, p = 0.1195). **c** There was no significant difference in motor performance between hNB001- and saline-treatment adult mice (Saline, n = 10 mice, hNB001, n = 10 mice, Student’s t-test, t _(18)_ = 0.5280, p = 0.6040). **d** The two squares on the left are representative traces showing the movement of hNB001- and saline-treatment mice in the open field test. The pink box is the central area and the green is the peripheral area. There was no significant difference in motor performance and anxiety-related behavior of the open field test after a single oral administration of hNB001 in adult mice, compared with the saline group (Saline, n = 10 mice, hNB001, n = 10 mice, Student’s t-test, total distance, t _(18)_ = 0.6008, p = 0.5555; Time in center, t _(18)_ = 0.2326, p = 0.8187; Number of center entries, t _(18)_ = 0.4884, p = 0.6311). **e, f** There was no significant difference in hind paw withdrawal to von Frey filaments (**e)** and response latency of the hot plate test **(f)** after a single oral administration of 10 mg/kg hNB001 in aged mice, compared with the saline group (Mechanical withdrawal, saline, n = 9 mice, hNB001, n = 10 mice, Student’s t-test, t _(17)_ = 1.328, p = 0.2017; Hot plate, saline, n = 8 mice, hNB001, n = 8 mice, Student’s t-test, t _(14)_ = 0.8348, p = 0.4179). **g** There was no significant difference in motor performance between oral administration of hNB001 and saline in aged mice (Saline, n = 10 mice, hNB001, n = 10 mice, Student’s t-test, t _(18)_ = 1.058, p = 0.3039). **h** The two squares on the left are representative traces showing the movement of hNB001- and saline-treatment mice in the open field test. The pink box is the central area and the green is the peripheral area. There was no significant difference in motor performance and anxiety-related behavior of the open field test after a single oral administration of hNB001 in aged mice, compared with the saline group (Saline, n = 9 mice, hNB001, n = 8 mice, Student’s t-test, total distance, t _(15)_ = 0.8076, p = 0.4319; Time in center, t _(15)_ = 1.390, p = 0.1862; Number of center entries, t _(15)_ = 1.363, p = 0.1959)

Previous studies have shown that NB001 had an analgesic effect in mice with chronic pain models [7, 27], and to test whether hNB001 has potential effects on normal mice, we performed behavioral tests of nociception, motor function, and anxiety-like behavior. In mechanical withdrawal threshold measurement, we found that a single oral administration of hNB001 (10 mg/kg) did not produce any significant effects in adult or aged mice (Fig. 4a, e). We also examined thermal nociception by hot plate test in hNB001-treated mice. Oral administration of hNB001 did not produce a significant difference in the response latency in adult or aged mice (Fig. 4b, f). These results suggest that a single oral application of hNB001 does not affect acute nociceptive responses in adult or aged mice. Next, we tested the effects of hNB001 on two different motor function tests, the RotaRod motor test and the open field test. The RotaRod motor test showed no significant difference in response latency between hNB001 and saline groups in adult or aged mice (Fig. 4c, g). In the open field test, hNB001 did not cause any significant changes in the total distance traveled (Fig. 4d, h). These results indicate that a single administration of hNB001 does not affect motor function in adult or aged mice. In addition, in the open field test, hNB001 did not cause any significant changes in the time spent of the center or the number of center entries in adult or aged mice (Fig. 4d, h), illustrating that hNB001 does not affect anxiety-like behavior in adult or aged mice.

### Effects of continuous application of hNB001 for 30 days on weight, nociception, motor function, and anxiety-like behavior

**Fig. 5:**
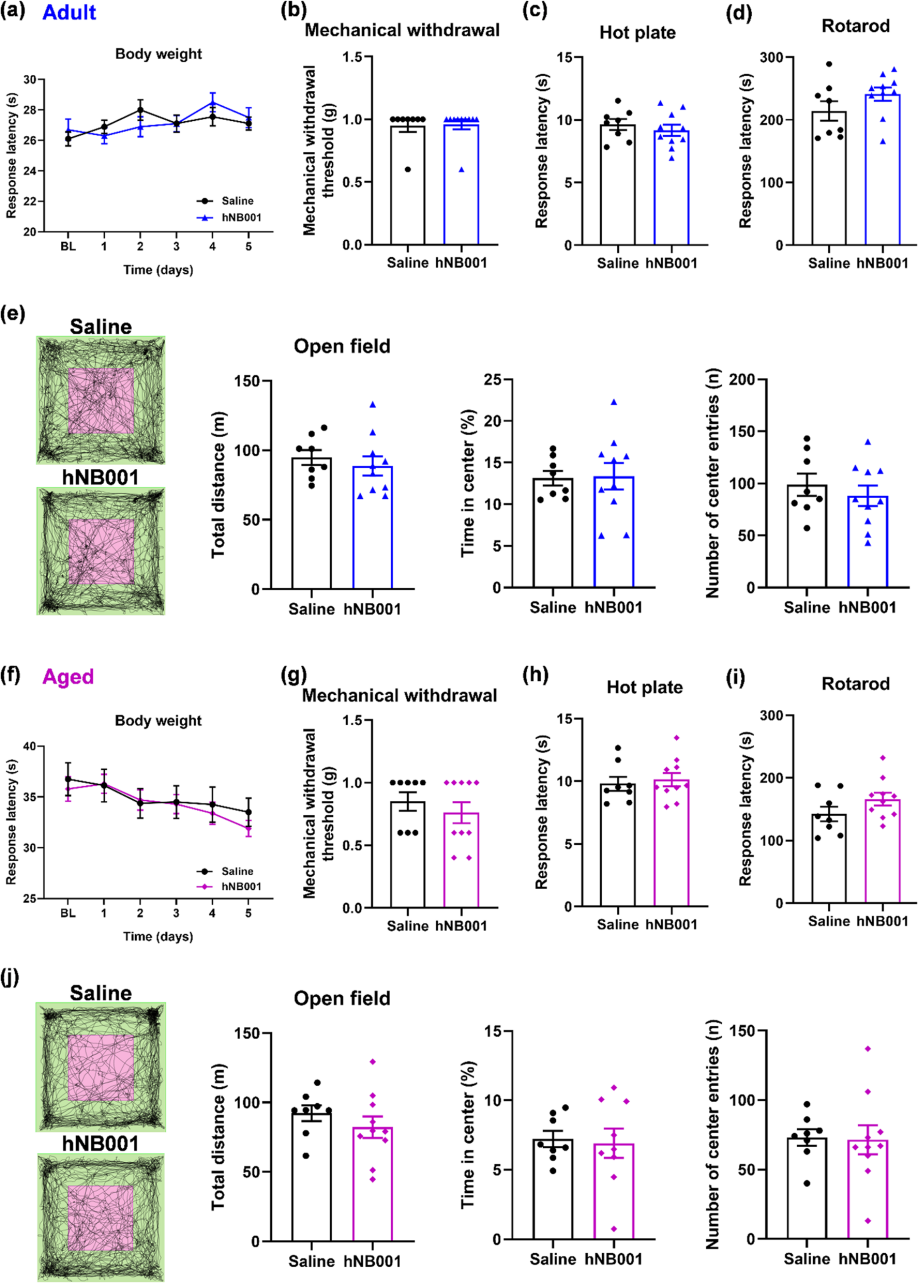
Effects of continuous administration of hNB001 on weight, nociception, motor, and anxiety-like behaviors in mice. **a** Continuous oral administration of 10 mg/kg hNB001 did not affect the weight of adult mice (Saline, n = 10 mice; hNB001, n = 10 mice, Two-way ANOVA, F _(1,18)_ = 0.0152, p = 0.9031). **b, c** Continuous oral administration of hNB001 did not affect hind paw withdrawal to von Frey filaments (**b**) and response latency of the hot plate test (**c**) in adult mice (Saline, n = 8 mice, hNB001, n = 10 mice, mechanical withdrawal, Student’s t-test, t _(16)_ = 0.1582, p = 0.8762; Hot plate, Student’s t-test, t _(16)_ = 0.7155, p = 0.4846). **d** Continuous oral administration of hNB001 did not affect motor performance in adult mice (Saline, n = 8 mice, hNB001, n = 10 mice, Student’s t-test, t _(16)_ = 1.460, p = 0.1638). **e** The two squares on the left are representative traces showing the movement of hNB001- and saline-treatment mice in the open field test. The pink box is the central area and the green is the peripheral area. Continuous oral administration of hNB001 did not affect motor performance and anxiety-related behavior of the open field test in adult mice (Saline, n = 8 mice, hNB001, n = 10 mice, Student’s t-test, total distance, t _(16)_ = 0.6770, p = 0.5081; Time in center, t _(16)_ = 0.1241, p = 0.9028; Number of center entries, t _(16)_ = 0.7239, p = 0.4796). **f** Continuous oral administration of 10 mg/kg hNB001 did not affect the weight of aged mice (Saline, n = 8 mice; hNB001, n = 10 mice, Two-way ANOVA, F _(1,16)_ = 0.1211, p = 0.7324). **g, h** Continuous oral administration of hNB001 did not affect hind paw withdrawal to von Frey filaments (**g**) and response latency of the hot plate test (**h**) in aged mice (Saline, n = 8 mice, hNB001, n = 10 mice, mechanical withdrawal, Student’s t-test, t _(16)_ = 0.7895, p = 0.4413; Hot plate, Student’s t-test, t _(16)_ = 0.4190, p = 0.6808). **i** Continuous oral administration of hNB001 did not affect motor performance in aged mice (Saline, n = 8 mice, hNB001, n = 10 mice, Student’s t-test, t _(16)_ = 1.539, p = 0.1434). **j** The two squares on the left are representative traces showing the movement of hNB001- and saline-treatment mice in the open field test. The pink box is the central area and the green is the peripheral area. Continuous oral administration of hNB001 did not affect motor performance and anxiety-related behavior of the open field test in aged mice (Saline, n = 8 mice, hNB001, n = 10 mice, Student’s t-test, total distance, t _(16)_ = 0.9894, p = 0.3372; Time in center, t _(16)_ = 0.2430, p = 0.8113; Number of center entries, t _(16)_ = 0.1349, p = 0.8944)

To further test the effects of long-term administration of hNB001, we performed a range of behavioral tests in adult and aged mice after oral administration of hNB001 (10 mg/kg) for 30 days. First, by measuring the weight of mice before and after oral administration of hNB001 or saline, we found that hNB001 did not affect the body weight of adult or aged mice (Fig. 5a, f). Results of mechanical withdrawal threshold measurement and hot plate test showed that continuous administration of hNB001 did not affect mechanical and thermal nociception in adult or aged mice (Fig. 5b, c, g, h). In addition, there was no significant difference in response latency of the RotaRod test or total distance traveled of the open field test between hNB001- and saline-treated adult mice (Fig. 5d, e), and there was no difference in aged mice (Fig. 5i, j). These results indicate that continuously treated hNB001 does not affect motor functions in adult or aged mice. Moreover, hNB001 did not produce a significant difference in the time spent of the center or the number of center entries in the open field test in adult or aged mice (Fig. 5e, j). In summary, continuous oral administration of hNB001 does not affect body weight, nociception, motor function, or anxiety-like behavior in adult or aged mice.

## Discussion

Cumulative evidence has strongly suggested that AC1 contributes to spinal and cortical excitation which is critical for chronic pain and related emotional changes [1–3, 20]. Neuron-selective AC1 is proposed to be a novel target for the treatment of different forms of chronic pain [7, 20, 31]. In the present study, we showed that hNB001 did not affect different forms of fear memory in adult mice. These findings are consistent with previous reports by our groups [7, 27] as well as other researchers [23]. Furthermore, we found that a single or long-term oral administration of hNB001 also did not affect nociception, motor functions, or anxiety-like behavior in both adult and aged mice on physiological conditions. These results strongly suggest that NB001/hNB001 is safe in both animals and humans [24]. We also found that hNB001 may enhance the relearning ability of the trace fear extinction in aged mice in the current study, confirming a previous genetic report that overexpression of AC1 lead to the reduction of memory in aged mice [23]. Together, these results indicate that inhibiting AC1 activity in aged animals may be beneficial for cognitive function, in addition to alleviating chronic pain.

### AC1 deletion or inhibition did not affect memory in adult mice

Previous studies have shown that AC1 deletion or inhibition did not affect recent and remote fear memory in adult mice (Table 1). For example, AC1 KO mice exhibited normal recent contextual and auditory fear memory [15]. Pharmacological experiments also showed that AC1 inhibitor NB001 did not affect recent contextual fear memory in male or female mice [7, 27]. Similarly, our results showed that hNB001 did not affect recent auditory fear memory in adult mice. Moreover, it was reported that AC1 KO mice showed normal remote contextual fear memory retrieval 5 weeks after training [32]. In the present study, we found that hNB001 also did not affect the retrieval of remote auditory fear memory in adult mice 30 days after training. Fear extinction is a type of inhibitory learning that suppresses learned fear memories [29]. A previous study showed that AC1 KO adult mice exhibited normal remote contextual memory extinction 3 weeks after training [32]. Consistent with this, our results showed that hNB001-treated adult mice exhibited normal remote auditory memory extinction 30 days after training. Taken together, genetic deletion or pharmacological inhibition of AC1 did not affect recent or remote fear memory, or fear extinction in adult mice. It is possible that the learning and memory of adult mice may be compensated for by other isoforms of ACs, such as AC8, and other key signaling proteins, including CaMKII, CaMKIV, PKC, and PKA [17, 18, 33].

**Table 1:** Effects of AC1 deletion or pharmacological inhibition on behaviors in adult mice

Potential effects	AC1 KO	NB001	hNB001 (present study)	Gabapentin
Contextual/Auditory fear memory	No change [15]	No change [7, 27]	No change	N/A
Remote fear memory/extinction	No change (5 w/3 w) [32]	N/A	No change (30 d/30 d)	N/A
Other types of memory	Spatial memory: impaired [44]; Trace fear memory: no change [42]	N/A	Trace fear memory: no change; Trace fear extinction: improved	Spatial memory: improved [45]; Inhibitory avoidance: a single treatment: improved [46], repeated treatment: impaired [40]
LTP	ACC: blocked [5] Hippocampus: no change [15]	ACC: blocked [7, 27] Hippocampus: no change [7]	ACC: blocked [24]	ACC: no change [37] Hippocampus: no change [36]
Acute pain	No change [6]	No change [7, 27]	No change [24]	No change [38, 39]
Chronic neuropathic pain	Alleviated [6]	Alleviated [7, 27]	Alleviated [24]	Alleviated [47]
Motor function	No change [34]	No change [7, 27]	No change	No change [38]
Anxiety-like behavior	No change [35]	No change [7, 27]	No change	Improved [45]

### Nociception, motor, and emotional responses in adult mice

Genetic deletion of AC1 or systematic application of NB001 inhibited mechanical allodynia in chronic pain model mice [6, 7, 27], suggesting that AC1 may be a potential target for the treatment of chronic pain. However, neither genetic deletion of AC1 nor NB001 affected behavioral responses to mechanical stimuli and acute noxious thermal stimuli [6, 7, 27]. Consistent with this finding, we found that hNB001, which can be used for the treatment of chronic pain in humans, also did not affect acute nociceptive responses to mechanical stimuli and noxious thermal stimuli in naive mice. Previous studies showed that AC1 KO or NB001-treated mice exhibited normal motor function and anxiety-like behavior [34, 35]. In the present study, hNB001 did not affect motor function and anxiety-like behavior in naive adult mice (Table 1).

Gabapentin is commonly prescribed for pain and can reduce chronic neuropathic pain. It has been reported that gabapentin did not alter the LTP of the hippocampus and ACC, but decreased basal synaptic transmission of the ACC [36, 37]. Our previous results showed that NB001/hNB001 blocked the induction of ACC LTP, but did not affect basal synaptic transmission of the ACC and LTP of the hippocampal [7, 24, 27]. These studies suggest the analgesic effects of gabapentin and NB001/hNB001 may be through different mechanisms. A single application of gabapentin did not affect acute nociception and motor in naive mice [38, 39], but long-term application impaired inhibitory avoidance memory and produced adverse reactions (Table 1) [40, 41]. The effects of long-term use of NB001/hNB001 on the behaviors of adult mice had never been examined before, and we tested behavioral changes in adult mice after 30 consecutive days of oral administration of hNB001. We found that long-term administration of hNB001 did not affect nociception, motor function, or anxiety-like behavior in adult mice. Our results provide strong evidence that hNB001 can be safely used for long-term treatment in the future.

### AC1 contributes to memory loss in aged animals

Increasing evidence shows that the expression of AC1 mRNA is downregulated in the hippocampus of aged mice [22]. It has been reported that forebrain AC1 overexpression in aged mice impaired spatial memory [23]. Consistent with this finding, we found that hNB001 enhanced the relearning ability of aged mice in trace fear extinction in the current study. It suggests that the decrease in AC activity during aging of mice may be an adaptive mechanism required to maintain learning and memory functions. In our study, compared to the control group, aged mice treated with hNB001 showed no difference in freezing during the beginning of trace fear test phase, and then produced a significant reduction. Although we believe that hNB001 enhanced the relearning of trace fear in aged mice, we cannot rule out the possibility that hNB001 inhibits trace fear memory recall. This needs to be further explored in future studies. It is well known that LTP is a major cellular model of learning and memory [1]. NB001/hNB001, as a selective AC1 inhibitor, blocked LTP of the ACC but not the hippocampal [7, 27]. In addition to the ACC, the hippocampus and its related nuclei may also contribute to the regulation of fear extinction. hNB001 may affect other signaling pathways (such as protein phosphatases through PKA) to contribute to relearning. Future studies are clearly needed to investigate this new mechanism.

Trace fear conditioning and test can examine attention-demanding associative learning and memory in animals. A previous study reported that gene deletion of AC1 or AC8 does not affect the acquisition and expression of trace fear in adult mice [42]. Similarly, our results showed that the AC1 inhibitor hNB001 did not affect the acquisition or expression of trace fear in aged mice. Consistent with the results in adult mice, hNB001 did not affect recent or remote auditory fear memory in aged mice. In addition, a single or continuous administration of hNB001 did not affect body weight, acute nociceptive response, motor functions, or anxiety-like behavior in aged mice. These support the safe application of hNB001 in elderly patients in the future.

### NB001 and the safety of both humans and animals

Previous genetic studies found that deletion of AC1 did not produce significant impairments in learning and memory [15], acute pain [6], or anxiety-like behaviors [35]. We infer that this is mainly due to three major reasons: (i) AC1 is selectively expressed in neurons, and it is not found in other non-neuronal tissues such as heart, kidney, and liver; (ii) AC1 is activated in an activity-dependent manner, and it plays major roles in physiological conditions; (iii) Key brain physiological functions, such as learning and memory, can be compensated by other isoforms of ACs such as AC8 as well as other protein kinases that also contribute to learning-related plasticity [16–18]. Consistently with these hypotheses, recent studies in both animals and healthy humans found that NB001 or hNB001 produced no significant side effects [7, 24, 27]. In the present study, we found that neither single nor continuous administration of hNB001 affected nociception, motor function, and anxiety-like behavior in adult and aged mice. These lay a good basis for the clinical application of the hNB001 in the future.

## Materials and methods

### Animals

Adult (8 to 12 weeks old) male C57BL/6 mice were purchased from the Experimental Animal Center of Xi’an Jiaotong University. Aged (16 to 20 months old) male C57BL/6 mice were purchased from Beijing Vital River Laboratory Animal Technology Co., Ltd. All animals were randomly housed under an artificial 12 h light/dark cycle with food and water provided ad libitum. All experimental protocols were approved by the Ethics Committee of Xi’an Jiaotong University.

### Drug application

hNB001 was obtained from Forevercheer Holding Ltd. Co. (Hong Kong, China), it was dissolved in saline. Referring to the published studies about NB001/hNB001 [24, 27], high doses of hNB001 (10 mg/kg and 30 mg/kg), which can produce a significant analgesic effect on mice of chronic pain models, were finally selected as the oral doses of mice.

### Trace fear memory

Trace fear training and test were performed in an isolated shock chamber (Shanghai Vanbi Intelligent Technology Co., Ltd.). The CS was an 80-db white noise for 15 s, the US was a 0.75-mA electric footshock for 0.5 s. For trace fear training, mice were acclimated for 60 s, and were conditioned by 10 CS-trace-US-ITI trials (trace, 30 s; ITI, 210 s) after oral administration hNB001 (30 mg/kg) or saline twice a day for 7 days. One day after training, mice were acclimated for 60 s and received 10 CS–ITI trials in a novel chamber for testing after oral administration hNB001 or saline for 45 min [26]. All data were video recorded and analyzed by Tracking Master software (Shanghai Vanbi Intelligent Technology Co., Ltd.). During training and testing, the average freezing for each ITI was analyzed. Bouts of 1.0 s were used to define freezing.

### Auditory fear memory and extinction

The experiment consisted of four phases, fear training, recent fear memory test, remote fear memory test, and fear extinction. Experiments were performed in an isolated shock chamber (Shanghai Vanbi Intelligent Technology Co., Ltd.). The CS was an 85-db tone at 2800 Hz for 30 s, the US was a 0.75-mA electric footshock for 2 s. For auditory fear training, mice were acclimated for 2 min, and received the 3 CS-US pairing and ITI (a 30 s CS and a 2 s US starting at 28 s; ITI, 30 s). One day after training, mice were acclimated for 2 min and received 3 CS-ITI trials in a novel chamber to test for recent fear memory after oral administration hNB001 (10 mg/kg) or saline for 45 min [6]. The mice were then treated with hNB001 (10 mg/kg) or saline orally twice a day for 30 days. Thirty days after training, mice were acclimated for 2 min and received 3 CS–ITI trials in a novel chamber to test for remote fear memory [28]. After 24 h of remote fear memory test, the fear extinction was performed once a day for three days. 45 min before extinction, the mice received hNB001 (10 mg/kg) or saline orally. During extinction, CS-ITI was repeatedly presented 12 times without the shock US delivery (ITI, 30 s) in a novel chamber [30]. All data were video recorded and analyzed by Tracking Master software (Shanghai Vanbi Intelligent Technology Co., Ltd.). During tests, average freezing for CS and ITI were analyzed. During extinction, the average freezing for each CS-ITI was analyzed. Bouts of 1.0 s were used to define freezing.

### Mechanical withdrawal threshold measurement

The mechanical hypersensitivity was determined with von Frey filaments (Stoelting; Wood Dale, Illinois) using the up-down method as previously reported [43]. Mice were individually placed into a plastic cage with wire mesh floors and allowed to acclimate for 30 min before testing. The von Frey filaments were applied perpendicularly to the plantar surface of the paw until it buckled slightly and was held for 3–6 s. Positive responses include licking, biting, and sudden withdrawal of the hind paw. An initial filament force of 0.4 g was applied to the mice. If a negative response occurred, the filament force was incrementally increased until a positive response was obtained. If the positive response occurred, the filament force was decreased until a negative result was obtained. Rest for 3–5 min after each positive reaction. This up-down method was repeated until five changes in behavior were determined. Recorded the value of each positive and negative response.

### Hot plate test

Mice were placed in the behavior room and allowed to acclimate for 30 min before testing. The mouse was placed on a hot plate at 55 ± 1 °C. The latency time in the first positive reaction of the hind paws was recorded. Positive responses include lifting, licking, shaking, and jumping. The cut-off time is 20 s to avoid tissue damage. The test was repeated three times with an interval of 30 min. The average of the three reactions was used for the final latency to response [27].

### Rotarod test

To test motor functions, the Rotarod test was performed as previously described [27]. 1 h before the test, the mice were trained to stay on the rotating drum for 1 min at a constant acceleration of 16 rpm. When tested, the Rotarod was set to accelerate from 4 to 40 rpm over a 5 min period. 5 min is set as the maximum time per session. The test was repeated three times with an interval of 5 min. The average of the three reactions was used for the final latency to the response.

### Open-field test

To record locomotor activity, the open-field test was performed as previously described [27]. Mice were placed in an open field (40 × 40 × 30.5 cm) and allowed to explore freely for 30 min. Define the 20 × 20 cm in the center of the open field as the center zone and the rest as the periphery zone. Total distance, the number of center entries, and time spent in the center were recorded and analyzed (tracking master v3.0 system).

### Body weight change measurement

During the 30 days of continuous oral administration of hNB001, the mice were weighed before and every week after oral administration until remote fear memory and fear extinction were measured.

### Statistical analysis

All data were reported as the means ± standard error of the mean (SEM). Data were analyzed and plotted with GraphPad Prism 8.0. For comparison between the two groups, statistical significance was assessed using unpaired Student’s t-test. For comparison among three or more groups, statistical significance was assessed using two-way ANOVA. In all cases, *p < 0.05 was considered statistically significant.

## Data Availability

The datasets used and/or analyzed during the current study are available from the corresponding author on reasonable request.
